# The efficacy of Equine Assisted Therapy intervention in gross motor function, performance, and spasticity in children with Cerebral Palsy

**DOI:** 10.3389/fvets.2023.1203481

**Published:** 2023-08-15

**Authors:** Alexandra N. Stergiou, Sanna Mattila-Rautiainen, Dimitrios N. Varvarousis, Meropi Tzoufi, Panagiota Plyta, Alexandros Beris, Avraam Ploumis

**Affiliations:** ^1^Division of Surgery, Department of Physical Medicine and Rehabilitation, University of Ioannina Medical School, Ioannina, Greece; ^2^Ioannina Therapeutic Riding Center, Ioannina, Greece; ^3^Sports and Exercise Medicine, Biomedicine, University of Eastern Finland, Kuopio, Finland; ^4^Department of Anatomy, University of Ioannina Medical School, Ioannina, Greece; ^5^Division of Child’s Health, Department of Paediatrics, University of Ioannina Medical School, Ioannina, Greece; ^6^Division of Surgery, Department of Orthopaedics, University of Ioannina Medical School, Ioannina, Greece

**Keywords:** Cerebral Palsy, equine assisted therapies, gross motor function, gross motor performance, spasticity

## Abstract

**Purpose:**

To evaluate the efficacy of Equine Assisted Therapy in children with Cerebral Palsy, in terms of gross motor function, performance, and spasticity as well as whether this improvement can be maintained for 2 months after the end of the intervention.

**Methods:**

Children with Cerebral Palsy participated in this prospective cohort study. The study lasted for 28 weeks, of which the equine assisted therapy lasted 12 weeks taking place once a week for 30 min. Repeated measures within the subject design were used for the evaluation of each child’s physical performance and mental capacity consisting of six measurements: Gross Motor Function Measure-88 (GMFM-88), Gross Motor Performance Measure (GMPM), Gross Motor Function Classification System (GMFCS), Modified Ashworth Scale (MAS) and Wechsler Intelligence Scale for Children (WISC III).

**Results:**

Statistically significant improvements were achieved for 31 children in Gross Motor Function Measure and all its subcategories (*p* < 0.005), also in total Gross Motor Performance Measure and all subcategories (p < 0.005). These Gross Motor Function Measure results remained consistent for 2 months after the last session of the intervention. Regarding spasticity, although an improving trend was seen, this was not found to be statistically significant.

**Conclusion and implications:**

Equine Assisted Therapy improves motor ability (qualitatively and quantitatively) in children with Cerebral Palsy, with clinical significance in gross motor function.

## 1. Introduction

Cerebral Palsy (CP) is a permanent, non-progressive encephalopathy that occurs in the brain during its development, before, during the birth, and up to 2 years after the birth (1–3). Children with CP have atypical posture and gait patterns due to abnormal muscle tone, reduced control of their muscles, static and dynamic imbalance, incoordination and asymmetry between agonist and antagonist muscles and poor equilibrium reflexes (4, 5). The main target of any therapeutic intervention is to enable patients to carry out daily activities and participation as independently as possible (International Classification of Functioning d230) (6–8).

In equine assisted therapy (EAT) the movement of the horse is utilized to improve functional and sensory limitations of individuals with movement disorders (9, 10). During EAT the muscles strengthen and the range of motion of joints is improved. Also, their stability, the coordination of the movement, the synergy of muscles, the displacement of weight shift and the control of the balance (11, 12) are improved while the oscillation of the patient is reduced due to its effort to maintain posture on the horseback (13–15). EAT also enhances the stability of the hip and trunk with hip and pelvic flexibility (16, 17).

Studies have shown that EAT is beneficial for children with CP for motor function abilities (13, 18–22), standing (13, 20, 23, 24) and sitting balance (25–27), gait parameters (13, 19, 28), the reduction of spasticity (23, 29–31), the symmetry of muscle activity (32), the joint range of motion (29) as well as in psychosocial domains and quality of life (18, 19, 21, 33, 34). The common outcome measures in the above studies were the Gross Motor Function Measure (GMFM) and Pediatric Balance Scale (PBS).

Furthermore, the duration of the positive effect of the EAT after its termination is questionable, having not been extensively investigated. In the literature, only two studies (35, 36) using GMFM found that participants had positive results in their gross motor function that was maintained from seven to 10 weeks, following the completion of the intervention.

This study aimed to investigate the effect of EAT on gross motor function, performance, and spasticity in children with CP in terms of GMFM, Gross Motor Performance Measure (GMPM) and Modified Ashworth Scale (MAS). This study also aims to investigate whether these improvements continue to exist after a two-month follow-up from the completion of the intervention.

## 2. Materials and methods

This prospective cohort study was registered in the clinical trials database (NCT01621984 Unique Protocol ID: 274/21-9-2011) and was approved by the Scientific Committee [12/24-8-2011 (θ.17)] and Administration Board (38/3–102,011 θ.33) of the University Hospital of Ioannina. All procedures performed in studies involving human or animal participants were in accordance with the ethical standards of the institutional and/or national research committee and with the 1964 Helsinki Declaration and its later amendments or comparable ethical standards.

### 2.1. Data collection

#### 2.1.1. Participants

Participants were sought within the registry of the Department of Physical Medicine and Rehabilitation and the Pediatrics Department of the General University Hospital of Ioannina, as well as through the non-profit organization “MERIMNA.” Informed consent was signed by the parents or caregivers after informing the purposes of the study and were assured the confidentiality of the personal data. Assessment and selection of children who met the inclusion criteria followed as outlined below. This study conforms to all CONSORT and STROBE guidelines and reports the required information accordingly.

#### 2.1.2. Inclusion criteria

The Inclusion criteria included: (1) children aged from 3 to 18 years old with CP; (2) written parental consent; (3) children with adequate range of motion to sit astride on the horse (participants should also have at least partial head control).

#### 2.1.3. Exclusion criteria

Exclusion criteria included: (1) unregulated epileptic seizures; (2) any musculo-skeletal disorder which could be aggravated by the motion of the horse; (3) allergy to the dust of the riding arena; (4) previous experience in EAT (5) botulinum toxin injections in any muscle during the last six months and (6) any surgery within a year prior to the study.

### 2.2. Instruments

#### 2.2.1. GMFM-88

The Gross Motor Function Measure is the most common quantitative outcome measure used for children with CP (37, 38) to evaluate a change occurring over time in gross motor ability after various clinical interventions (37, 38). It quantitatively measures gross motor function, an activity a child can do (GMFM-88) or a level of motor ability achieved (GMFM-66) (38). GMFM-88, which was used for the present study, includes 88-point assessment criteria, being distributed across 5 categories: (Α) lying and rolling, (Β) sitting, (C) crawling and kneeling, (D) standing and (E) walking, running & jumping. Each category is comprised of several elements graded from 0 to 3 units (0 = the child is not able to start an activity and 3 = the child is able to fully complete the activity). It has been studied for its reliability and validity (37–39).

#### 2.2.2. GMPM

The Gross Motor Performance Measure assessed the quality of movement, which means how well an activity is completed. It has been designed to be used in combination with GMFM (40, 41). It is a criterion-based observational measure evaluating five different aspects of quality of movement: alignment, stability, coordination, weight shift and dissociation over 20 GMFM items (40, 41). It additionally has been studied for its reliability and validity (41, 42).

#### 2.2.3. MAS

The Modified Ashworth Scale is a measure of resistance to passive stretch which has been studied for its reliability and validity. A six-point numerical scale (0, 1, 1+, 2, 3, 4) grade spasticity from zero to four, with zero being no resistance and four being a joint rigid in flexion or extension (43, 44). While resistance and passive movement from the hip joint were both measured from five repetitions.

#### 2.2.4. Measuring procedure

Gross motor function and performance, as well as spasticity of all participants who were included in the study, were assessed. All participants were categorized cognitively, with Wisc III, and motorically, with the GMFCS. The total number of children was subdivided into two subgroups, the first included children with severe deficits and the second included children with mild and moderate deficits, according to their cognitive or/and functional capacity. This was completed to identify changes in cognitive and gross motor capacity (progress of the functionality) within these two groups of children.

For each patient, six measurements took place using the GMFM scale and the MAS. The GMPM scale was measured at two different time points, before and after the intervention (Table 1). Successive assessments of the children were carried out by two independent researchers (AP, DV) who were experienced in the use of the assessments and were blinded to the results of previous assessments. The evaluators of Wisc III were child psychiatrists who were blinded to the study and this assessment took place in State Pediatrics Educational Center in Ioannina.

**Table 1:** Time points for the assessment of GMFM, GMPM and MAS.

Time points for GMFM and MAS	
**Assessments**	**Time point**
*Assessment 1	8 weeks before the start of the intervention
	4 weeks before the intervention
	Just before the planned intervention
Assessment 2	6 weeks after the start of the intervention
Assessment 3	12 weeks after the start of the intervention (at the end of the intervention)
Assessment 4	8 weeks after the end of the intervention
Timepoints for GMPM	
Assessment 1	Just before the planned intervention
Assessment 2	12 weeks after the start of the intervention (at the end of the intervention)

* Assessment 1 calculated as the average value of the 3 measurements performed before the intervention.

GMFM, gross motor function measure.

GMPM, gross motor performance measure.

MAS, modified Ashworth scale.

### 2.3. Intervention

The equine assisted therapy lasted for 3 months (12 weeks) each session consisting of 30 min of exercise on the horseback taking place every week at the Ioannina Therapeutic Riding Center, Greece. Participants continued to receive their conventional rehabilitation program throughout the pre-and post-intervention as well as during the intervention.

Three horses, trained for therapy purposes, of varying sizes were used to match the sizes of the participants. Two qualified professionals in EAT carried out the intervention which was individualized to the needs of every child. Trained side-walkers ensured the safety of the mounted child and horse leaders followed the instructions from the EAT practitioner for the individualized walking rhythm of the horse. A soft saddle pad with a vaulting girdle was used for the children to be able to perceive the horse’s temperature and transmitted movement more easily (13, 45). Adjustable stirrups in the vaulting girdle were used for performing exercises, such as sitting and standing up (45).

All children wore protective riding helmets. Children according to their ability mounted the horse from a mounting ramp with assistance, independently or were passively placed on the horseback by the professional leading the session. The EAT sessions were carried out depending on the children’s classification of performance ability according to GMFCS and mental capacity according to Wisc III.

The horse was being led in straight lines, in circles, or a “figure of eight” between cones and serpentines and the child was sitting on the horseback with the eyes open or closed. The horse’s gait also varied (moderate to fast walking and trotting) (45, 46). One goal for the participant was to be able to sit independently, with good alignment and symmetry (47).

The ones that were able to follow directions, either because their mental capacity (Wisc III) allowed them to do so or because of their functional capacity (GMFCS I, II, III) or as an outcome of a combination of the above, played a more active role in their therapy and performed more complicated activities.

Each child, depending on motor ability, actively or passively changed position while on the horse (i.e., sitting astride or laying back or in front on the neck of the horse or sitting sideways on the horse) (48, 49). Different body positions on the moving horse ensured that the child would receive multiple vestibular stimuli (46, 48, 49) Stirrups were used (50) so that the child would be able to lift himself and sit back again (48, 51) to strengthen the lower limbs and to improve in shifting the centre of gravity and balance (48).

In order to attain the objectives, set for the EAT intervention, a series of exercises were performed. Exercises that were masked to a form of play were easier to perform. The exercises performed consisted of catching and tossing a ball, throwing rings on the cones, throwing bean bags on the basket, and searching for hidden objects on the horse by catching and tossing a ball to a basket from a moving horse the eye-hand coordination, planning, timing and needed force to perform the task were trained. Hiding objects underneath a saddle pad was aimed to train body orientation and problem-solving.

Children of classification IV and V in GMFCS in combination with respective Wisc III classification, children presenting serious and severe mental disabilities, received passive mobilization of their body on the horseback, directional changes, gradually building up the stimuli depending on their needs and limitations.

To enable active participation with performance wherever possible, a passive or active-assisted approach was applied for exercises of the trunk and extremities (reaching, weight shifting), while on the horse, to increase the range of motion. An effort was made for their active participation wherever this was possible.

### 2.4. Statistical analysis

Statistical analyses were conducted using Stata 14.1 (StataCorp, College Station, TX, United States). A longitudinal analysis was performed for the GMFM. Univariate and multivariate mixed-effects linear regression models were used (time series analysis was used based on single and multiple linear mixed-effects models with individuals as random effects). In the univariate models, the time of measurement was the primary variable. The comparison for the subgroup was done with paired t-test. The results were considered significant at the level 0.05.

The comparison of the GMPM scale values was done in a univariate manner with paired *t*-tests and in a multivariate way using longitudinal analysis methods.

To compare the values of the MAS at different time points, Fisher’s exact test was used. In the multivariate models, the results were adjusted for possible confounding variables, such as gender, age, and assessment based on Wisc III and the GMFCS level for the aforementioned three types of assessment tools. Data on MAS, GMFM and its subcategories were available for six-time points. The first three took place before the intervention. For increased accuracy with respect to the initial measurement of MAS and GMFM, the mean of the three GMFM measurements prior to intervention was used.

## 3. Results

Thirty-five children fulfilled the initial inclusion criteria and were included in the study. One child withdrew immediately after the first assessment; another one after the second; and two others after the third assessment, all prior to the start of the intervention because the caregiver did not see a benefit to the intervention. Finally, 31 children participated in the EAT intervention (Table 2).

**Table 2:** Demographic data as a whole and per GMFCS level.

Characteristic	Total		GMFCS I		GMFCS II		GMFCS III		GMFCS IV		GMFCS V	
	*N* (31)	%	*N*	%	*N*	%	*N*	%	*N*	%	*N*	%
Sex												
Male	18	58.06	4	50	3	37.5	4	66.67	3	75	4	80
Female	13	41.94	4	50	5	62.5	2	33.33	1	25	1	20
WISC III												
Normal	10	32.26	6	75	2	25	2	33.33	0	0	0	0
Low average	1	3.23	1	12.5	0	0	0	0	0	0	0	0
Mild	1	3.23	0	0	0	0	1	16.67	0	0	0	0
Moderate	3	9.68	1	12.5	1	12.5	0	0	1	25	0	0
Sever	9	29.03	0	0	2	25	0	0	2	50	5	100
Profound	7	22.58	0	0	3	37.5	3	50	1	25	0	0
Type of CP												
Hemiplegia	2	6,45	2	25								
Diplegia	12	38,71	5	62,5	6	75	1	16,67	1	25		
Quadriplegia	17	54,84	1	12,5	2	25	5	83,33	3	75	5	100

	**N**	**Mean (SD)**	**N**	**Mean (SD)**	**N**	**Mean (SD)**	**N**	**Mean (SD)**	**N**	**Mean (SD)**	**N**	**Mean (SD)**
Age	31	10.39 (5.07)	8	7.13 (3.72)	8	11.75 (5.7)	6	11.17 (5.31)	4	12 (5.89)	5	11.2 (4.6)
Weight	31	35.16 (17.51)	8	27.5 (13.09)	8	47 (20.3)	6	35.67 (18.73)	4	37.25 (17.8)	5	26.2 (9.98)
Height	31	1.29 (0.27)	8	1.23 (0.22)	8	1.4 (0.35)	6	1.29 (0.18)	4	1.26 (0.35)	5	1.21 (0.27)

GMFCS, gross motor function classification system.

WISC, Wechsler intelligence scale for children.

From the 31 children, two subgroups were divided according to their mental or/and functional capacity. Eleven children with severe deficits, and 20 children with mild and moderate deficits. The term severe impairment referred to children with a low score in WISC III (profound and severe, that usually are not able to follow rules and communicate) or/and are classified as Level III, IV, V in GMFCS. Children with severe deficits needed more assistance from the therapist in contrast with children with mild and moderate impairments that they could be more active in the intervention due to their higher cognitive and motor function level (Table 2).

### 3.1. GMFM-88

**Figure 1:**
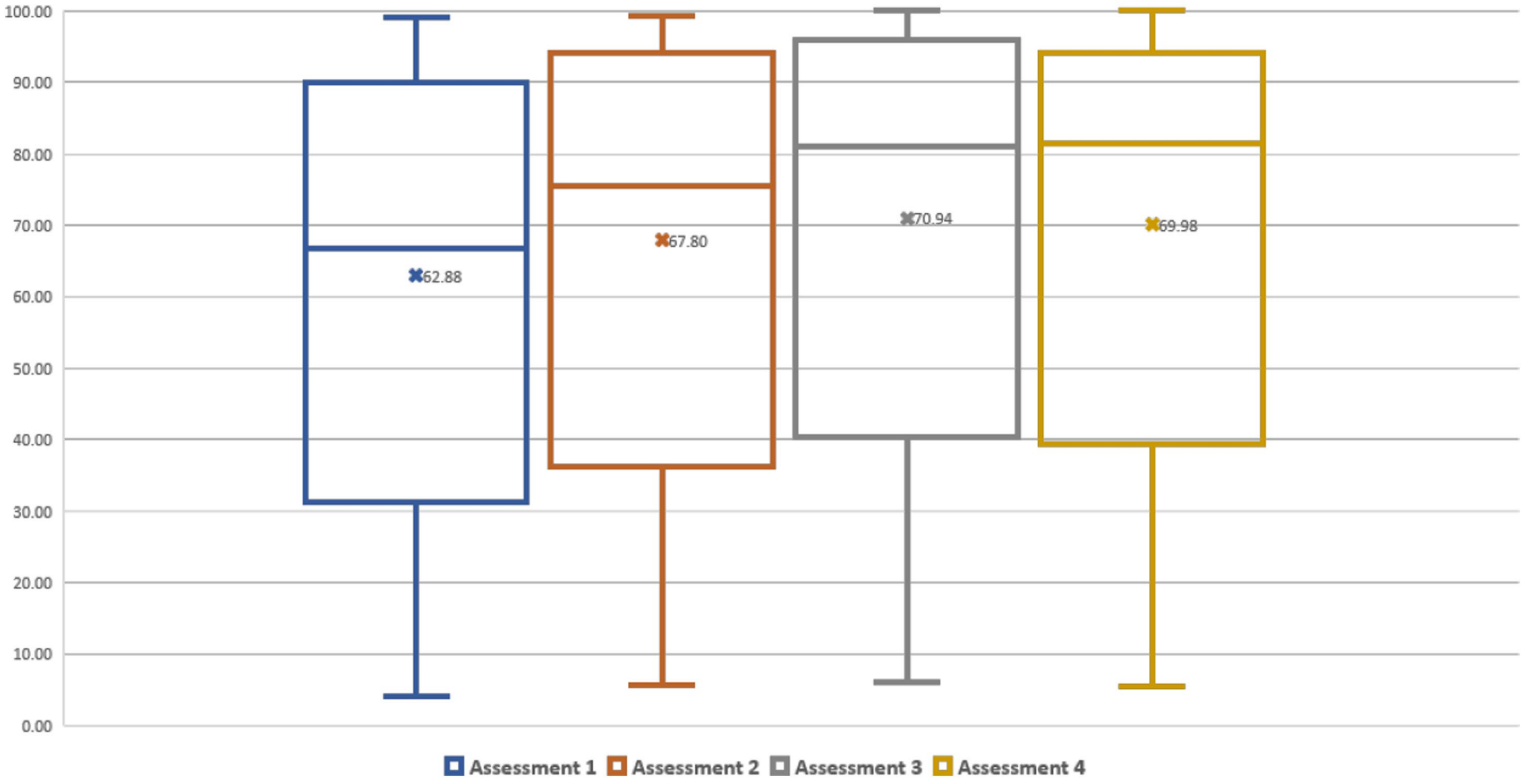
Diagram of mean GMFM of the 4 assessments.

Of 31 children, 29 participated in the last assessment (assessment no 4) that took place 2 months after the end of the intervention. The total score of GMFM increased significantly (*p* < 0.0001) after 6 weeks (assessment no 2) (mean difference = 4.92) as well as after 12 weeks (assessment no 3) of the intervention (mean difference = 8.05) (Table 3; Figure 1). Nevertheless, a statistically significant decrease (*p* = 0.0217) in the total GMFM score was observed between the 3rd third (at the 12th week, the end of the intervention) and the fourth 4th assessment (2 months after the end of the intervention). However, the total GMFM in the 4th assessment was still significantly better (*p* < 0.005) than the 1st assessment and about the same as the 2nd assessment (mean difference = 2.18). Statistically significant improvements were also observed in all subcategories of GMFM (A-D) but not in subcategory E (walking running and jumping) of non-ambulatory children (level V of GMFCS) (*p* > 0.005). A greater improvement of the total score was found for children classified as GMFCS III (13.65), then IV (10.61), then ΙΙ (9.98), V (4.26) and lastly Ι (3.03) (differences between assessments 1st and 3rd).

**Table 3:** Comparison of the GMFM and dimensions (A, B, C, D, E) between the different time points (assessments 2, 3, 4) and the initial measurement (assessment 1).

Characteristic	Ν	Total (*Ν* = 31)		Value of *p*	
		Mean (SD)	Mean difference (SE*)	Univariate model	Multivariate model**
GMFM, A					
Assessment 1	31	81.54 (22.19)	0	–	–
Assessment 2	31	86.4 (19.36)	4.86 (1.02)	<0.0001	<0.0001
Assessment 3	31	89.41 (18.22)	7.87 (1.02)	<0.0001	<0.0001
Assessment 4	29	88.03 (19.93)	6.31 (1.06)	<0.0001	<0.0001
GMFM, B					
Assessment 1	31	74.68 (29.84)	0	–	–
Assessment 2	31	78.92 (28.95)	4.25 (1.17)	0.0003	0.0003
Assessment 3	31	82.15 (28.91)	7.47 (1.17)	<0.0001	<0.0001
Assessment 4	29	81.73 (28.19)	5.27 (1.21)	<0.0001	<0.0001
GMFM, C					
Assessment 1	31	61.93 (39.42)	0	–	–
Assessment 2	31	67.28 (38.42)	5.35 (1.06)	<0.0001	<0.0001
Assessment 3	31	69.12 (38.23)	7.19 (1.06)	<0.0001	<0.0001
Assessment 4	29	69.05 (37.26)	5.79 (1.10)	<0.0001	<0.0001
GMFM, D					
Assessment 1	31	53.27 (36.78)	0	–	–
Assessment 2	31	58.23 (37.78)	4.96 (1.30)	0.0001	0.0001
Assessment 3	31	62.61 (37.74)	9.35 (1.30)	<0.0001	<0.0001
Assessment 4	29	60.68 (36.85)	7.03 (1.35)	<0.0001	<0.0001
GMFM, E					
Assessment 1	31	44.01 (34.88)	0	–	–
Assessment 2	31	48.16 (36.36)	4.15 (0.99)	<0.0001	<0.0001
Assessment 3	31	51.48 (37.47)	7.47 (0.99)	<0.0001	<0.0001
Assessment 4	29	50.2 (36.81)	6.26 (1.02)	<0.0001	<0.0001
GMFM, Total					
Assessment 1	31	62.88 (30.83)	0	–	–
Assessment 2	31	67.8 (30.44)	4.92 (0.71)	<0.0001	<0.0001
Assessment 3	31	70.94 (30.24)	8.05 (0.71)	<0.0001	<0.0001
Assessment 4	29	69.98 (30.08)	6.38 (0.73)	<0.0001	<0.0001

* Compared to the pre-intervention value.

** The multivariate model has been weighted for gender, age, level of WISC III and GMFCS level.

GMFM, gross motor function measure.

### 3.2. GMPM

Gross motor performance measure was measured of 29 participants. Distribution across the GMPM scale and its subcategories, for two different time points is presented in Table 4. A statistically significant increase in GMPM and all its subcategories was achieved (Table 4). Children classified as level I (8.17) on the GMFCS showed greater improvement in the total score, followed by levels V (6.66), IV (6.25), III (5.58) and lastly, II (4.93) (differences between assessments 1st and 3rd).

**Table 4:** Comparison of the GMPM and its subcategories, before and after the intervention.

Scale	Before the intervention *N* = 29 Mean (SD)	After the intervention *N* = 29 Mean (SD)	Mean difference (SE)	Paired *t*-test *p*	Multivariate model*
Dissociated movement	51.69 (30.38)	56.4 (28.38)	4.71 (1.55)	0.005	0.0019
Coordination	52.82 (22.51)	60.56 (24.45)	7.74 (1.93)	0.0004	<0.0001
Alignment	52.46 (18.05)	57.24 (21.75)	4.78 (1.73)	0.0101	0.005
Weight shift	42.87 (18.64)	49.93 (21.9)	7.06 (1.50)	0.0001	<0.0001
Stability	56.39 (21.73)	64.81 (21.21)	8.42 (1.85)	0.0001	<0.0001
Total	51.25 (21.25)	57.66 (22.39)	6.42 (1.12)	<0.0001	<0.0001

* The multivariate model has been weighted for gender, age, level of WISC III and GMFCS level.

### 3.3. MAS

Nineteen of the participants had spasticity and three of them did not participate at the last assessment 2 months after the end of the intervention. Ashworth scale values of the different time points are presented in Table 5. A decrease in spasticity is seen over the time points, but it was not found to be statistically significant per Fisher’s exact test criterion (*p* = 0.350).

**Table 5:** Modified Ashworth scale results.

Grades of MAS	Assessment 1	Assessment 2	Assessment 3	Assessment 4
	*N* (%)	*N* (%)	*N* (%)	*N* (%)
Missing	0 (0)	0 (0)	0 (0)	3 (9.86)
0	12 (38.71)	12 (38.71)	13 (41.94)	12 (38.71)
1	1 (3.23)	1 (3.23)	2 (6.45)	1 (3.23)
1+	4 (12.9)	6 (19.35)	4 (12.9)	7 (22.58)
2	6 (19.35)	4 (12.9)	10 (32.26)	3 (9.68)
3	7 (22.58)	7 (22.58)	1 (3.23)	4 (12.9)
4	1 (3.23)	1 (3.23)	1 (3.23)	1 (3.23)
Total	31 (100)	31 (100)	31 (100)	31 (100)

Fisher’s exact test, *p*-value = 0.350.

MAS - modified Ashworth scale.

### 3.4. Results of subgroups

All participants, regardless of mild–severe deficits, demonstrated statistically significant improvement in the GMFM (mean difference = 7.16 and 9.67 respectively) and GMPM (mean difference 6.53 and 6.19 respectively) (*p* < 0.05) (Table 6). No statistical significance was observed between the two groups.

**Table 6:** Mean (SD) total GMFM and GMPM in subcategories of children with 1. mild and moderate deficits and 2. severe deficits.

Subgroups	Mild and moderate deficits *Ν* = 20			Severe deficits *Ν* = 11			Difference between groups		
	Difference before-after	Standard Error	value of p	Difference before-after	Standard error	value of *p*	Difference	Standard error	*p*-value
GMFM total	7.16	1.21	<0.001	9.67	2.02	0.007	2.51	2.02	0.260
GMPM total	6.53	1.54	0.0005	6.19	1.51	0.0027	0.34	2.40	0.887

GMFM, gross motor function measure.

GMPM, gross motor performance measure.

### 3.5. Minimal clinically important differences (MCID)

According to the literature (52), clinically important improvement was observed (average low-value differential >1.29 and high value >3.99) in all GMFM analyses between the initial assessment (assessment no 1) and the 12th-week assessment (assessment no 3), regarding the total number of the children (8.06), but also in the subgroups of children with mild and moderate deficits (7.16) and children with severe deficits (9.67). The same was observed between the initial and the final assessment. This is a high-power study (100.00 and 95.86%, respectively, for average low differential and average high differential MCID) according to the post-hoc power estimation for Minimal Clinically Important Differences.

### 3.6. Adverse events

There were no adverse events related to the intervention. None of the participants suffered any injury or had any other complication during the study.

## 4. Discussion

This prospective study aimed to assess the effectiveness of EAT intervention in children with CP. The aforementioned results state, that the participants demonstrated improved GMFM scores that met the criteria of MCID at the last follow-up. All groups show statistical significance (*p* < 0.005) between the assessments (Table 4). An important note is that the results of the intervention show, that there is no statistical difference in the outcomes between the CP subgroups (mild and moderate vs. severe). As it is not correct to compare unsimilar groups we can see significant improvement in both subgroups, stating that the objectives for the rehabilitation were achieved regardless of the level of CP. The results for spasticity showed improvement but were not statistically significant.

In the literature, many studies have shown statistically significant improvement in some subcategories of GMFM and total score of GMFM-66 and GMFM-88 depending on the classification (level Ι to ΙV in GMFCS) (13, 20, 35, 47, 49, 50, 53). In our study, statistically significant improvement was observed in all subcategories and the total score of GMFM-88, but, as was expected, there was no statistically significant improvement in subcategory E (walking, running and jumping) of non-ambulatory children (level V of GMFCS).

In two other studies (35, 36), GMFM measurements took place at 7 and 10 weeks following the completion of the intervention, respectively, and found that positive results of GMFM were maintained (no statistically significant difference was noticed from the termination of intervention to the last follow-up). Similarly, in our study, the significant improvement of GMFM was maintained 8 weeks after the completion of the intervention, which was also clinically significant (according to MCID). This means that the increase of GMFM of patients being rehabilitated with EAT (following equine assisted exercises) was leading to better functional abilities.

Regarding GMPM, the current study showed that EAT intervention improved the quality of movement of children with motor dysfunctions. Similar studies in the international literature using GMPM, investigate the benefits of different types of therapeutic exercise with heterogeneous results (54–56).

In the study of MacKinnon et al. (21) children who were able to cooperate better (due to their mental capacity and functional skills) showed increased motor development. Based on our results using GMFM and GMPM and according to GMFCS level, it was observed that in quantitative measurement of motion (GMFM) children classified as II and III improved more compared to other subcategories, while children classified as I and V improved less. The opposite happens concerning the quality of movement (GMPM). A possible theory could be that EAT intervention benefits more children with mild and moderate functional deficits in gross motor function, while children with independent functionality or severe motor disorders are mainly benefited in terms of the quality of movement. Probably children with independent functionality (level I) as well as with severe motor disorders (V) cannot give statistically significant results in contrast to mild and moderate motor deficits (II, III) where the intervention seems to give statistically significant results.

While statistically significant differences were found in our measures, this does not necessarily translate to clinically important differences. Minimal Clinically Important Differences (MCID) provide the threshold for determining if clinically important differences take place before and after an intervention (57). Our study has shown the clinical significance of the change in gross motor function of children by the equine assisted therapy intervention. In the study of Davis et al. (33) both the statistical and clinical significance of gross motor function changes were not proven results, while in a review study by Little et al. (58), the clinically meaningful effect of hippotherapy on gross motor function in the short term was small.

Regarding spasticity and MAS, one randomized trial (59) and one meta-analysis (60) showed a statistically significant improvement in adductor spasticity (32, 59) and generally in the muscles of the pelvis and lower limbs (29, 61, 62). Nevertheless, in another study (63), results were similar to ours, since no statistically significant improvement had been observed concerning adductor spasticity. Antunes et al. (64) noticed an improvement in adductor spasticity when horse walking and trotting were included. It is worth mentioning that the benefits of this intervention were short-term (32, 59–62, 64). This is shown by the fact that measurements in these studies were made just before and after the treatment was completed (32, 61, 64), in contrast to our study, where spasticity was measured at a pre-determined appointment after the intervention. The above studies are possibly based on the fact that prolonged muscle stretching for a period of 10–30 min is effective in reducing spasticity and may last up to 35 min after exercise completion (65). It has also been mentioned that spasticity improvement has been maintained up to at least 4 days (62). The fact that in our study final assessments for each patient took place in a period of 1–5 days after the end of the intervention, may hide the possible beneficial immediate effect of EAT on spasticity.

### 4.1. Strengths and limitations

The strength of the study is the relatively large number of participants in comparison to other studies (10, 11, 17, 60, 66–68), as well as the participation of children of various functional levels and with a diversity of motor dysfunctions. It also categorized the results in children with different functional levels. The intervention was led by different professionals than those conducting the assessments and were blinded. Another strength was, that the horses with staff stayed the same for the children throughout the intervention.

A disadvantage of our study was that children varied greatly in functional classification in all subcategories according to GMFCS and Wisc III. So, in children of lower classification in Wisc III, communication and cooperation were difficult. We may have had more significant improvement with an intervention, which would have lasted several months longer or the conditions were designed for the children to participate more frequently in the therapy. This research, as others (47, 49, 50), uses the same children as a control group before and after the intervention, instead of other participants who would not have gone through any EAT as in a typical control group. This may be initially seen as a study limitation, but in reality, it may also be a sensitive way of detecting even the slightest therapeutic changes (49), since the development of each child with CP may vary. The children continued to receive their conventional therapies throughout the intervention period and follow-up. Even though the strength of the statistical analysis for the modified Ashworth scale changes is low, the results are valid for our sample.

## 5. Conclusion

The findings of this study support that EAT may improve gross motor function and performance in children with CP, even 2 months after the end of the intervention. These findings should be reinforced with more research, as many clinically significant results were found.

## Data availability statement

The original contributions presented in the study are included in the article/supplementary material, further inquiries can be directed to the corresponding authors.

## Ethics statement

The studies involving human participants were reviewed and approved by the Scientific Committee [12/24-8-2011 (θ.17)] and Administration Board (38/3–102,011 θ.33) of the University Hospital of Ioannina. Written informed consent to participate in this study was provided by the participants’ legal guardian/next of kin.

## Author contributions

AS: conceptualization, formal analysis, investigation, methodology, visualization, and roles and writing – original draft. SM-R: data curation, methodology, software, and writing – review and editing. DV: conceptualization, investigation, resources, software, visualization, roles and writing – original draft, and writing – review and editing. MT: conceptualization, investigation, project administration, and supervision. PP: data curation, investigation, and resources. AB: formal analysis, software, supervision, and writing – review and editing AP: conceptualization, formal analysis, investigation, methodology, project administration, supervision, validation, visualization, and writing – review and editing. All authors contributed to the article and approved the submitted version.

## Funding

This work was funded by the Ann Kern-Godal’s grant (No:11297) Unifor, Norway. One of the authors (SM-R) holds a shared doctoral researcher position at the University of Eastern Finland.

## Conflict of interest

The authors declare that the research was conducted in the absence of any commercial or financial relationships that could be construed as a potential conflict of interest.

## Publisher’s note

All claims expressed in this article are solely those of the authors and do not necessarily represent those of their affiliated organizations, or those of the publisher, the editors and the reviewers. Any product that may be evaluated in this article, or claim that may be made by its manufacturer, is not guaranteed or endorsed by the publisher.

## Abbreviations

CP: Cerebral Palsy
EAT: Equine Assisted Therapy
GMFCS: Gross Motor Function Classification System
GMFM: Gross Motor Function Measure
GMPM: Gross Motor Performance Measure
MAS: Modified Ashworth Scale
Wisc III: Wechsler Intelligence Scale for Children, 3rd edition
